# Neuropsychiatric complications of traumatic brain injury

**DOI:** 10.1192/j.eurpsy.2021.674

**Published:** 2021-08-13

**Authors:** C. Fernandes Santos, A.B. Medeiros, R. Gomes, N. Descalço

**Affiliations:** Psychiatry And Mental Health Department, Hospital Garcia de Orta, E.P.E., Almada, Portugal, Portugal

**Keywords:** postconcussion, traumatic brain injury, neuropsychiatry

## Abstract

**Introduction:**

Traumatic brain injury (TBI) is a leading cause of morbidity and mortality, giving rise to a variety of neuropsychiatric syndromes associated with great functional impairments, chronic disability and poor quality of life. Depending on diagnostic criteria, 20-90% of victims of TBI develop at least one neuropsychiatric manifestation in the first month, and about 40% present at least three symptoms during three months, with higher incidence in females. Survivors of TBI are at increased risk for development of severe, long-term psychiatric disorders. The aetiology of these disturbances remains unclear.

**Objectives:**

To review current knowledge on the neuropsychiatric consequences associated with TBI.

**Methods:**

Non-systematic review of literature through search on PubMed/MEDLINE database for publications up to 2020, following the terms “traumatic brain injury” and “neuropsychiatry”.

**Results:**

Although the experience of neuropsychiatric symptoms may be temporary and may resolve in the acute period, many patients with TBI can experience psychopathology that is persistent or that develops in the post-acute period, regardless of injury severity. These symptoms can involve personality changes, psychosis, major depression, generalized anxiety disorder, post-traumatic stress disorder, maladaptive social behaviours, poor disability adjustment, reduced coping skills and cognitive impairment. Evidence remains insufficient to conclude the role of TBI-related neuropathological consequences in the development of post-TBI neuropsychiatric disorder. Non-organic factors are also implicated in its generation and maintenance.

**Conclusions:**

Neuropsychiatric sequelae are common following TBI. Several of these syndromes are amenable to treatment. Further investigations are required to better understand the mechanistic aetiology of these conditions and the effectiveness of therapeutic modalities.

